# Sleep duration and adiposity in older adolescents from Otago, New Zealand: relationships differ between boys and girls and are independent of food choice

**DOI:** 10.1186/1475-2891-12-128

**Published:** 2013-09-14

**Authors:** Paula ML Skidmore, Anna S Howe, Maria A Polak, Jyh Eiin Wong, Alex Lubransky, Sheila M Williams, Katherine E Black

**Affiliations:** 1 Department of Human Nutrition, University of Otago, PO Box 56, Dunedin 9054, New Zealand; 2 Department of Psychology, University of Otago, PO Box 56, Dunedin 9054, New Zealand; 3 School of Healthcare Sciences, Faculty of Health Sciences, Universiti Kebangsaan Malaysia, Kuala Lumpur 50300, Malaysia; 4 Department of Preventive and Social Medicine, University of Otago, PO Box 56, Dunedin 9054, New Zealand

**Keywords:** Sleep, Body composition, Adolescents, New Zealand

## Abstract

**Background:**

While relationships between sleep and BMI have been extensively studied in younger children the effect of sleep duration on adiposity in adolescents, who are undergoing rapid growth periods, is less well known. There is also a lack of consistent evidence on the role of sleep on other measures of adolescent body composition which may be more reflective of health than BMI in this age group. Previous research investigating whether these relationships differ between sexes is also inconsistent. Therefore the objective of this study was to investigate relationships between sleep duration and multiple body composition measures in older adolescents and to investigate if these relationships differ between boys and girls.

**Methods:**

A web-based cross-sectional survey and anthropometric measurement of 685 adolescents (mean age 15.8 years) from 11 schools in Otago, New Zealand. Height and weight were measured by trained researchers and fat mass and fat-free mass were estimated using bio-impedance. Generalised estimating equations were used to examine associations between sleep duration and the following body composition measures: BMI, waist circumference (WC), waist-to-height ratio (WHtR), fat mass index (FMI), and fat-free mass index (FFMI). Analyses were adjusted for ethnicity, deprivation, the number of screens in the bedroom and fruit and vegetable consumption.

**Results:**

When data from all participants were analysed together, no significant relationships were seen between sleep duration and any body composition measure but significant sex interactions were seen. An hour increase in average nightly sleep duration in boys only was associated with decreases of 1.2% for WC, 0.9% for WHtR, 4.5% for FMI and 1.4% for FFMI in multivariate models. Similar results were seen for weekday and weekend night sleep duration.

**Conclusions:**

Sex specific factors may play a role in relationships between sleep and body composition in older adolescents. The results in boys were most pronounced for FMI, a measure of total adiposity, which suggests that insufficient sleep in adolescent boys may affect fat mass more than lean mass and that the use of measures such as BMI may result in an under-estimation of relationships.

## Background

High rates of overweight and obesity are common in many adolescent populations in Western countries and rates in New Zealand are among the highest in the world [1,2]. Data from the 2008/09 New Zealand Adult Nutrition Survey [2] show that over one third of those aged 15–18 years were overweight or obese. Excess weight in childhood and adolescence may have lasting effects throughout the lifecourse including increased risk of type two diabetes, hypertension and coronary heart disease later in life [3]. Short sleep duration has been identified as one possible cause of overweight and obesity [4-6]. Many negative outcomes have been associated with insufficient sleep that are also related to overweight and obesity, such as changes in the appetite controlling hormones leptin and ghrelin [7,8] as well as impaired glucose tolerance [9].

It is thought that adolescents need just over nine hours of sleep per day [10,11] as this amount of sleep allows adolescents to deal with the high demands of physical, emotional and sexual development [6]. Results of a recent meta-analysis suggest that older adolescents in particular are not getting enough sleep during weekdays and are sleeping over an hour longer at weekends to catch up on weekday sleep deficit [12]. This deficit of sleep on weekdays in particular is thought to be as a result of pubertal changes in circadian rhythms, which peak between the ages of 15 to 21 years, resulting in extended wakefulness during the evening coupled with having to wake early for school [13].

The relationship between short sleep duration and body mass index (BMI) has been investigated extensively in children and to a lesser degree in adolescents [4-6]. While shorter sleep duration has been consistently associated with BMI in children, the relationship between sleep and higher BMIs in adolescents is inconsistent [4-6]. A recent review [6] of studies investigating the relationship between sleep duration and excess weight in adolescents shows that the majority of studies use BMI as the only measure of adiposity. While BMI provides a relatively sensitive and specific surrogate measure of overweight and obesity in adolescents [14] it does not distinguish between fat and muscle tissue and it may be that insufficient sleep results in metabolic effects that influence body fat specifically [15]. It may also be the case that the relationship between sleep and body composition differs between adolescent boys and girls [16]. However, the majority of previous research has not investigated the role of sex interactions in the relationship between sleep and body composition [4,6].

Therefore the aim of this study was to investigate relationships between weekly, weekday and weekend day sleep duration and several measures of body composition (BMI, waist circumference, fat mass index, and fat-free mass index) using multivariate models to investigate if these relationships differ between adolescent boys and girls.

## Methods

### Study design and participants

The Otago School Students Lifestyle Survey Two (OSSLS2) study was a cross-sectional survey of 15 to 18 year olds from school years 11 to 13, who attended secondary schools in Dunedin and surrounding towns in Otago, New Zealand. In school term four (October to December) of 2010, 18 secondary schools from Otago were invited to take part in the OSSLS2 study. Individual student recruitment to OSSLS2 was undertaken in school terms one and two (February to June) of 2011. Randomly selected classes from years 11 to 13 from each school were invited to take part. The number of classes sampled at each school ranged from one per year group at the smaller schools to four per year group at the larger schools. In the week before the survey visit invited students were given packs containing separate information sheets and consent forms for students and their parents. Students were required to sign a consent form in order to participate, while parents were only required to provide opt-out consent on behalf of their child. The study was designed to be completed during one class period and consisted of an online survey and collection of anthropometric measurements. Teams of three or more trained research assistants conducted measurements at participating schools according to standard operating procedures. The study was approved by the University of Otago Human Ethics Committee.

### Questionnaire

The online questionnaire was conducted in each school’s computer labs. The survey included sections on demographics, food consumption, food habits and attitudes, and eating behaviours using questionnaires that were previously validated in similar populations where possible [17-20]. Ethnicity was categorised into three groups; Māori, Pacific or New Zealand European or Other (NZEO) in accordance with other national New Zealand surveys. Students self-reported their date of birth, age, sex, ethnicity and residential address. Socio-economic status (SES) was assessed using several indicators. Individual-level indicators used were household crowding and car ownership. Neighbourhood-level SES was assessed using the New Zealand Deprivation Index Score (NZDep06), which is derived from residential address and provides a measure of area-level deprivation [21]. The NZDep06 combines nine variables from the 2006 census that reflect eight dimensions of deprivation, including income, owning a house and access to a car. The deprivation index is an ordinal scale ranging from 1 (least deprived) to 10 (most deprived) and this variable was collapsed into five categories. School decile is a SES indicator of the school, and is based on the proportion of students at the school with low SES as defined by the student’s residential address. Decile 1 includes the 10% of schools with the highest proportion of students from low SES communities; decile 10 the lowest proportion. School decile was divided into ‘Middle’ (Deciles 5 to 8) and ‘High’ (Deciles 9 and 10).

Participants were asked to report what time they usually went to bed and what time they usually got up on schooldays and weekend days separately, as defined in the validated Sleep Habits Survey for Adolescents [18]. Schooldays and weekend days were not specified in the questionnaire but face validity of the questions was assessed in group interviews during survey pretesting. Results of this pretesting indicated that students recognised that the question “At the weekend what time do you usually go to bed?” referred to Friday and Saturday nights, whereas “At the weekend what time do you usually get up?” referred to Saturdays and Sundays (unpublished data). The following relevant sleep duration variables were calculated using this information: Average Sleep Duration (average nightly sleep over a week), Weekday Sleep Duration (average nightly sleep on weekdays only) and Weekend Sleep Duration (average nightly sleep on weekend days only). Average Sleep Duration was calculated using a ratio of five weekdays to two weekend days. The difference in sleep duration (Sleep Difference) between weekdays and weekends was also calculated. Experienced researchers piloted the survey questions and data collection methods for comprehension and acceptability in a sample of Dunedin students from the relevant age group. The online survey was also piloted to ensure it could be completed within one class period and refinements were made to the survey before data collection.

### Body composition measurements

Body composition measurements were taken in a nearby classroom. Height was measured with a calibrated portable stadiometer (University of Otago, New Zealand), with shoes and socks removed and head in the Frankfort plane. Waist circumference (WC) was measured at the midpoint between the lower costal margin and the level of the anterior superior iliac crest using a non-elastic tape (Seca, Germany) during mid-expiration. As WC measurements were taken under clothes and participants were asked to hold or tuck up their tops or shirts these measurements were taken in a private screened-off area of the room. Height and WC measurements were taken twice to the nearest 0.1 cm and if the two measurements differed by more than 0.5 cm a third measurement was taken.

A segmental bio-electrical impedance analysis (BIA) scale (Tanita, type BC-418, Japan) was used to measure weight to the nearest 0.1 kg, impedance and body fat percentage, fat mass and fat free mass. Fat mass and fat free mass estimates obtained from BIA measurements, using this particular scale, in New Zealand adolescents have been found to be highly correlated with DXA measurements, with correlations of 0.97 for fat mass and 0.96 for fat free mass [22]. This BIA model provides reliable results, with Technical Error of Measurement values of 0.13% for weight, 0.18% for fat free mass, 0.74% for fat mass and 0.33% for impedance (unpublished data). Participants with either embedded metal pins or plates or cardiac pacemakers, or who indicated they might be pregnant or could not otherwise undergo impedance were excluded from BIA measurements. Participants’ height was measured before BIA was undertaken so this information could be inputted into the scale. Participants were measured in light indoor clothing with bare feet and a standard clothing weight of 0.5 kg was used for clothing. Participants were asked to remove all jewellery and to empty their pockets before stepping onto the scale. Research staff checked that participants’ bare feet touched the metal plates and that no items of clothing were impeding this. Participants stood with feet on the metal plates and knees apart and with their arms down but slightly away from the body.

BMI was calculated as weight (in kilograms) divided by height squared (in meters). Age and sex specific BMI z-scores were calculated using the 2007 WHO method [23] and BMI values were categorized using the IOTF sex- and age-dependent cut-offs [24]. Because of the low prevalence of thinness and obesity in the current sample an “Overweight” variable was calculated for use in analyses by combining the thinness and normal weight categories into one category and overweight and obesity into another category. BMI IOTF category data was used only to describe population characteristics, whereas BMI z scores were used in the regression analyses. Fat mass index (FMI) was calculated by dividing fat mass (in kilograms) by height squared (in meters), fat-free mass index (FFMI) was calculated by dividing fat-free mass (in kilograms) by height squared (in meters) and waist-to-height ratio (WHtR) was calculated as WC (in centimeters) divided by height (in centimeters).

### Data handling and statistical analyses

Participants were excluded if they had incomplete demographic information, as this was the first section of the survey. Participants were also excluded if their responses indicated the survey was not completed properly. Examples of this included clicking patterns (e.g. selection of extreme left or right answers for all questions), contradictory responses to similar questions, or multiple unrealistic answer options. Only participants who had complete data for sleep duration and all body composition variables were included in the final analysis. Descriptive data were summarised as frequencies and percentages, means with standard deviations, or medians with inter-quartile ranges. Differences in demographics between BMI categories were investigated using Chi-squared (χ^2^) tests, while independent t-tests were used to examine differences in body composition between boys and girls, and sleep variables between boys and girls.

Regression analyses were performed using Gaussian family generalized estimating equations with robust standard errors, to allow for the clustering in data among students attending the same schools [25], to ensure results are representative of the population in the sampled area. Each analysis included a sleep variable (Average Sleep Duration, Weekday Sleep Duration, Weekend Sleep Duration or Sleep Difference) and a single body composition variable. The influence of confounding variables was assessed by fitting four models, with Model 1 adjusted for age and sex. As deprivation, ethnicity, food choice and television/computer/videogame screen use have both been found to be associated with body composition [6,26] model 2 was adjusted for age, sex, school decile, ethnicity, the number of screens in the particpant’s bedroom and whether the participant met national recommendations for fruit and for vegetable consumption (F&V recommendation). Model two was repeated using different individual measures of deprivation, including NZDep06, household crowding and car ownership, and different indicators of a healthy diet (frequency of consumption of fruit, vegetables, chocolates and confectionery, chips, crisps and soft drinks). As these different markers of SES or diet made no difference to effect sizes and school decile and F&V recommendation were the only two relevant variables where data was available for every participant these were used in the final analyses. Model 3 consisted of Model 2 plus the relevant sex by sleep variable interaction term. As differences in the relationship between sleep and overweight have been found between boys and girls [6], Model 4 was run separately in boys and girls and was adjusted for age, school decile, and ethnicity. Model results in tables are presented with no interaction terms. When a significant interaction was found this was indicated with a superscript in the table while the relevant models including the interaction terms were presented visually with figures. As some of the models were not linear in their association, all body composition variables except BMI were logarithmically transformed. Therefore, β coefficients are presented as a change in BMI z-score per hour increase in sleep, and as a percentage change in geometric mean per hour increase in sleep for all other body composition variables. All analyses were performed using the STATA statistical software package version 12SE (StataCorp LP, College Station, TX, USA).

## Results

### Descriptive analysis

**Figure 1:**
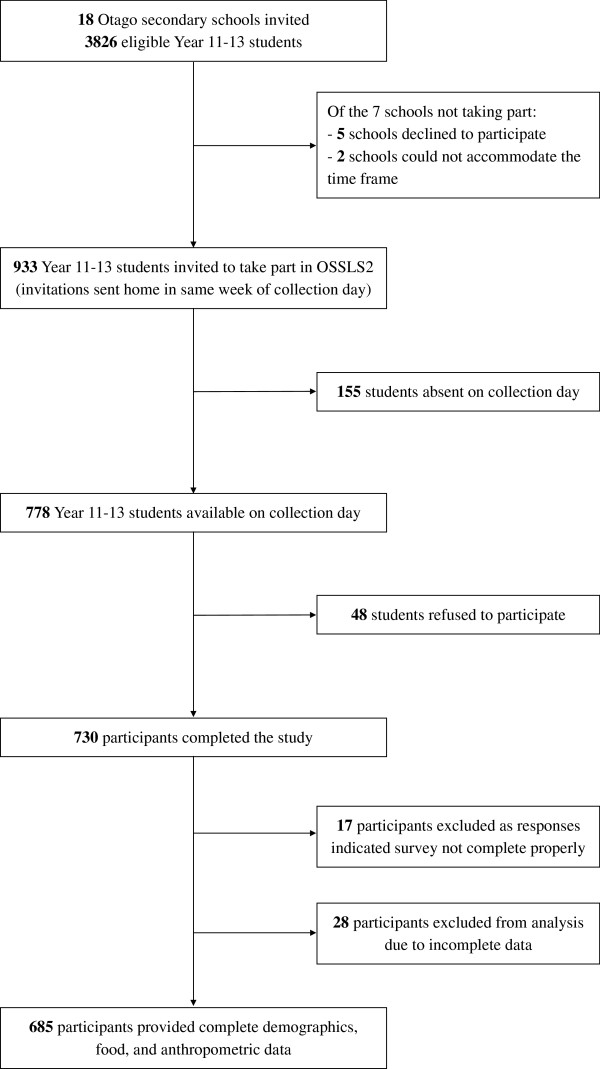
**Recruitment of schools and adolescents into the Otago School Students Lifestyle Survey Two (OSSLS2) study.** Not every class was invited into the study; the number of classes per school was dependent on the school year size at each school. This ranged from one class per year in smaller schools to four classes per year at larger schools. Reasons for exclusion from the final dataset included clicking patterns detected, consistently choosing contradictory options to similar questions or picking multiple unrealistic answer options.

Students from 11 out of the 18 invited schools took part in OSSLS2 (Figure 1). From a total pool of 933 eligible students from these schools, 788 of whom were available on the relevant school data collection day, 730 took part in the survey (student response rate of 78% of all those eligible, or 93% of those eligible who were not absent from school on the relevant data collection day). Participating students made up 25% of all year 11 to 13 secondary students in the entire Otago region of New Zealand. Complete sleep, demographic, F&V recommendation, screen and body composition data were available for 685 participants. The school deciles for the 11 participating schools ranged from 5 to 10. The mean age of participants was 15.8 (±0.9) years, 56% of the sample were male, 90% were of NZEO and 20% were overweight and 6% obese (Table 1). Sixty one percent of students were from schools with a decile rating of 9 or 10 (Table 1).

**Table 1:** Demographic characteristics of participants in the OSSLS2 by BMI classification

		**Total sample**		**BMI classification**			
				**Normal**		**Overweight**	
		**n**	**(%)**^**1**^	**n**	**(%)**^**1**^	**n**	**(%)**^**1**^
All		685		502	(73)	183	(27)
Sex							
	*Girls*	299	(44)	219	(73)	80	(27)
	*Boys*	386	(56)	283	(73)	103	(27)
Ethnicity							
	*NZEO*	616	(90)	461	(75)	155	(25)
	*Māori*	59	(9)	38	(64)	21	(36)
	*Pacific*	10	(1)	3	(30)	7	(70)
School year							
	*11*	304	(44)	219	(72)	85	(28)
	*12*	205	(30)	154	(75)	51	(25)
	*13*	176	(26)	129	(73)	47	(27)
School decile							
	*Middle*	267	(39)	185	(69)	82	(31)
	*High*	418	(61)	317	(76)	101	(24)
NZDep06							
	*1 (Least)*	283	(42)	208	(74)	75	(26)
	*2*	129	(19)	103	(80)	26	(20)
	*3*	114	(17)	78	(68)	36	(32)
	*4*	98	(15)	72	(73)	26	(27)
	*5 (Most)*	44	(7)	30	(68)	14	(32)

*OSSLS2* Otago School Students Lifestyle Survey Two, *NZEO* New Zealand European and Other, *NZDep06* New Zealand Deprivation Index.

^1^Row percentage, unadjusted for confounders.

Median sleep duration was 9 hours 11 minutes for weekdays and 10 hours at weekends, and differences in sleep duration were seen between boys and girls (Table 2). 17.7% of participants reported going to bed after 11.30 pm on weekdays and 30% reported going to bed after midnight at weekends (data not shown). Median difference in sleep time between weekends and weekdays was one hour fifteen minutes (IQR: 30 – 120 minutes) (Table 2). 23% of participants slept for two hours longer on weekend days compared to weekdays (data not shown). As expected there were differences in all body composition measures between boys and girls and those who were overweight also had less favourable levels of all other body composition measurements (data not shown).

**Table 2:** Daily time spent in bed (hours : minutes) by male and female participants in OSSLS2

	**All participants (n = 685)**		**Girls (n = 299)**		**Boys (n = 386)**	
	**Median**	**(IQR)**	**Median**	**(IQR)**	**Median**	**(IQR)**
Entire week	9:11	(8:34, 9:45)	9:17^a^	(8.37, 9:49)	9:08^a^	(8:34, 9:41)
Weekdays only	9:00	(8:15, 9:30)	9:00^a^	(8:15, 9:30)	9:00^a^	(8:15, 9:30)
Weekend days only	10:00	(9:00, 10:45)	10:00^a^	(9:00, 11:00)	10:00^b^	(8:45, 10:30)
Sleep Difference	1.15	(0:30, 2:00)	1:15^a^	(0:30, 2:00)	1:00^a^	(0:30, 2:00)

*OSSLS2* Otago School Students Lifestyle Survey Two.

Sleep difference = the difference in sleep hours per night between weekends and weekdays (Weekend - Weekday); For each sex group, values within a row with unlike superscript letters were significantly different (P < 0.05).

### Multivariate analysis

When data from boys and girls were analysed together in regression analyses no consistent significant effects were seen in Models 1 or 2 (Table 3), with the exception of FFMI, where results from Model 2 were approaching significance (*P* = 0.08 for Average Sleep Duration and *P* = 0.06 for Weekend Sleep Duration). When data were analysed separately for boys and girls no significant relationships were seen for girls. Significant negative relationships between Average Sleep Duration and Weekday Sleep Duration and all body composition measures were seen for boys (Table 3). An hour increase in Average Sleep Duration in boys was associated with decreases of 0.1 for BMI z-score, 1.2% for WC, 0.9% for WHtR, 4.5% for FMI and 1.4% for FFMI. Adjustment for ethnicity, school decile, F&V recommendation and number of screens in the bedroom made no meaningful difference to effect sizes in any model.

**Table 3:** Associations between sleep duration (hours per day) and body composition measures in participants of the OSSLS2

		**Total sample (n = 685)**				**Girls (n = 299)**		**Boys (n = 386)**	
		**Model 1**		**Model 2**		**Model 4**		**Model 4**	
		**Coef (95% CI)**	***P***	**Coef (95% CI)**	***P***	**Coef (95% CI)**	***P*-value**	**Coef (95% CI)**	***P***
BMI z-score^1^									
* Average sleep duration*		−0.06 (−0.15, 0.03)	0.20	−0.06 (−0.15, 0.04)	0.24	0.03 (−0.13, 0.20)	0.69	−0.11 (−0.20, -0.02)	0.02
* Weekday sleep duration*		−0.04 (−0.12, 0.04)	0.30	−0.04 (−0.12, 0.04)	0.33	0.05 (−0.11, 0.21)	0.56	−0.09 (−0.18, -0.01)	0.02
* Weekend sleep duration*		−0.03 (−0.08, 0.01)	0.17	−0.03 (−0.08, 0.02)	0.21	−0.02 (−0.12, 0.08)	0.72	−0.04 (−0.11, 0.03)	0.29
Waist circumference^2^									
* Average sleep duration*		−0.67 (−1.57, 0.23)	0.14	−0.68 (−1.61, 0.27)	0.16^3^	0.28 (−1.03, 1.60)	0.68	−1.17 (−2.01, -0.32)	0.01
* Weekday sleep duration*		−0.39 (−1.09, 0.32)	0.28	−0.41 (−1.15, 0.34)	0.28	0.33 (−1.14, 1.82)	0.66	−0.79 (−1.32, -0.26)	0.01
* Weekend sleep duration*		−0.51 (−1.10, 0.09)	0.09	−0.49 (−1.11, 0.13)	0.12	−0.05 (−0.92, 0.83)	0.91	−0.69 (−1.40, 0.02)	0.06
WHtR^2^									
* Average sleep duration*		−0.44 (−1.20, 0.32)	0.26	−0.42 (−1.24, 0.41)	0.32^3^	0.47 (−0.69, 1.64)	0.43	−0.92 (−1.64, -0.19)	0.01
* Weekday sleep duration*		−0.24 (−0.83, 0.36)	0.44	−0.21 (−0.86, 0.45)	0.54	0.42 (−0.80, 1.66)	0.50	−0.57 (−1.17, 0.03)	0.06
* Weekend sleep duration*		−0.36 (−0.86, 0.14)	0.16	−0.37 (−0.92, 0.18)	0.18	0.14 (−0.72, 1.01)	0.75	−0.60 (−1.19, 0.01)	0.05
Fat mass index^2^									
* Average sleep duration*		−1.87 (−6.06, 2.49)	0.40	−1.85 (−5.82, 2.29)	0.38^3^	2.40 (−2.08, 7.08)	0.30	−4.54 (−8.29, -0.63)	0.02
* Weekday sleep duration*		−1.11 (−4.51, 2.42)	0.53	0.82 (−3.98, 2.44)	0.62^3^	2.30 (−2.27, 7.09)	0.33	−2.98 (−5.81, -0.07)	0.05
* Weekend sleep duration*		−1.39 (−3.70, 1.98)	0.25	−1.77 (−4.14, 0.66)	0.15^3^	0.44 (−1.08, 1.98)	0.57	−2.84 (−5.71, 0.11)	0.06
Fat-free mass index^2^									
* Average sleep duration*		−0.88 (−1.74, 0.01)	0.05	−0.79 (−1.68, 0.10)	0.08^3^	0.16 (−1.04, 1.36)	0.80	−1.40 (−2.29, -0.50)	0.01
* Weekday sleep duration*		−0.50 (−1.15, 0.16)	0.15	−0.49 (−1.19, 0.21)	0.17	0.26 (−1.07, 1.61)	0.70	−1.00 (−1.68, -0.32)	0.01
* Weekend sleep duration*		−0.77 (−1.25, -0.10)	0.02	−0.56 (−1.14, 0.03)	0.06	−0.16 (−0.72, 0.41)	0.59	−0.75 (−1.53, 0.04)	0.06

WHtR = Waist Circumference (cm) to Height (cm) ratio; Model 1 = GEE regression adjusted for age and sex.

Model 2 = GEE regression adjusted for age, sex, school decile, ethnicity, F&V recommendation and number of screens in the bedroom.

Model 4 = GEE regression adjusted for age, school decile, ethnicity, F&V recommendation and number of screens in the bedroom.

^1^Expected change in mean BMI z-score for every hour increase in sleep.

^2^Expected percentage change in mean body composition for every hour increase in sleep.

**Figure 2:**
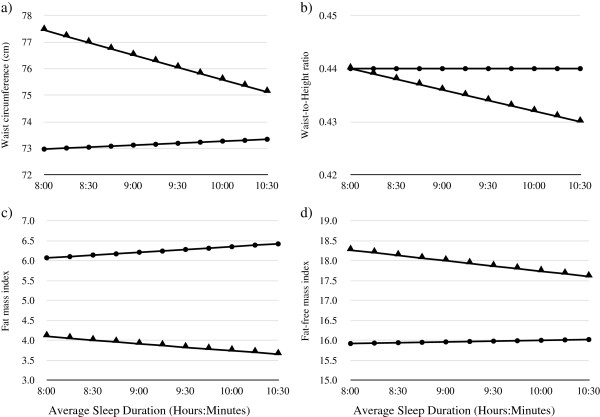
**Visual representation of the significant interaction between sex and average sleep duration for a) waist circumference; b) waist-to-height ratio; c) fat mass index; and d) fat-free mass index in boys and girls.** Figures display results for a 16-year old from a high decile school. Results are plotted for the fully adjusted model and include the relevant sex by sleep variable interaction term (model 3). (*P* < 0.05 for interaction in all four graphs) ● Girls ▲ Boys.

^3^Significant sex by sleep variable interaction observed in Model 3 (See Figure 2 for model results that include a significant interaction term for Average Sleep Duration).

Significant sex interactions were seen in relationships between Average Sleep Duration and WC, WHtR, FMI, FFMI as presented in Figure 2. The relationships between Weekend Sleep Duration and WC, WHtR, FMI and FFMI were of similar magnitude to those for Weekday Sleep Duration (Table 3) and no significant differences were seen between weekday to weekend Sleep Difference and any body composition variable (data not shown).

## Discussion

This study adds to the limited literature investigating relationships between measures of body composition other than BMI in older adolescent populations. In this cohort of older adolescents, we found that longer sleep duration was associated with lower levels of all body composition variables, including lower levels of fat free mass, in boys only. While significant results were seen for measures of central adiposity (waist circumference and waist-to-height ratio), the results were most pronounced for FMI, a measure of total adiposity. A one hour decrease in sleep on weeknights was associated with a 4.5% increase in FMI, after adjustment for age, deprivation, ethnicity, whether participants met recommendations for fruit and vegetable consumption and the number of screens in the bedroom. A major strength of this study is the use of several measures of body composition. The majority of previous studies in adolescents have used only BMI [6] and have categorised both sleep and BMI, with no consistent cut-points for short sleep or overweight being used, which makes direct comparisons of effect size between studies difficult. However, results from studies in younger children suggest that boys may be at greater risk of sleep-associated obesity than girls [4]. Our results for BMI are similar to the only other study in adolescents [16] where data were analysed in a similar way, where an hour decrease in sleep duration in adolescents boys was associated with a 0.1 increase in BMI z score.

Fewer studies have investigated the relationship between sleep and other measures of body composition and results from studies in adolescents have been inconsistent, with some studies finding significant results for both boys and girls and some only in boys [27]. However none of these studies have investigated these relationships in older adolescents, who have more autonomy with regards to lifestyle choices, including bedtimes and food choice, compared to younger children [27]. Even the most recent studies in this age group that have advantages such as using longitudinal rather than cross-sectional data [28], or have large, nationally representative samples [29] rely on using BMI measures only. While BMI is a measure of excess weight and not necessarily excess body fat, it has been shown to have good specificity [30], and may be a good proxy for fat mass in obese adolescents [23]. However, in normal-weight or overweight adolescents higher BMI may be more reflective of increased lean mass rather than fat mass [23]. For this reason BMI may not be a good proxy for body fat in this cohort, as a very small number of participants were obese. More consistent evidence has been found between sleep duration and more direct measures of fat mass as assessed by BIA or DXA (Duel-energy X-ray Absorbance) [27], however all of these previous studies have been in younger children. Therefore our results provide unique insight into the relationships between sleep and body composition in older adolescents.

Data were collected during the New Zealand summer and autumn, meaning that there were differences in daylight hours over the course of data collection. For example, in February, sunrise in the Otago region is at around 7 am and sunset at around 9 pm, whereas sunrise in June is at around 8 am and sunset at 5 pm. While it is possible that adolescent sleep patterns may be related to hours of daylight, previous research in adolescents suggests that factors other than hours of daylight, such as hormonal and physical changes, and shifting of circadian rhythms may potentially increase their risk of not gaining sufficient sleep [6,13].

In agreement with previous research [4,5] we found that the relationship between sleep and body composition was independent of ethnicity and deprivation. While ethnicity and deprivation were themselves significant in the models, they made no meaningful difference to effect sizes for relationships between sleep and body composition. However, the low numbers of Māori and Pacific participants and those from more deprived schools may have impaired our ability to detect the true effects of these variables. We also found that whether participants met recommendations for fruit and vegetable consumption and number of screens in the bedroom played no significant part in any relationships.

While we found significant negative relationships between sleep duration and all measures of body composition in boys, the largest effect sizes were seen for specific measures of fat and lean mass, rather than overall body composition. Our results show that while longer sleep duration was associated with both lower lean and fat mass, the effects were most pronounced for fat mass. Previous research in twin cohorts has shown that while an individual’s absolute amount of lean tissue may be determined in the fetal period, changes in the proportion of fat to lean tissue are influenced by their physical and social environment [31,32]. Changes in muscle mass can only be achieved by hypertrophy, whereas adipocytes can undergo both hypertrophy and hyperplasia, meaning that, in agreement with our findings, an individual’s environment may influence their fat mass more than their lean mass [31].

It is outside the scope of this study to determine the mechanisms by which short sleep duration affects body composition. Results from previous studies indicate that reduced sleep may increase dietary intake due to increased wakefulness leading to more opportunity to eat [4]. Hormonal changes may also play a role. Lower growth hormone levels have been observed in those who sleep less, which could result in adolescents not attaining their optimal genetically determined height, resulting in a higher BMI [33]. Sleep deprivation has also been shown to have adverse effects on leptin and ghrelin levels, leading to increased appetite [7,8] but we found no evidence that food choice, including increased frequency of high sugar and/or high fat foods, influences relationships between sleep and body composition in this cohort. Sleeping less may also lead to fatigue or changes in thermoregulation resulting in decreased energy expenditure [4].

A limitation of this study is that sleep data were based on self report and questions used in this study asked about time of going to bed and getting up and therefore our measures of sleep duration may reflect time at rest plus sleep time, rather than sleep per se. However, the questions used in this survey have been validated against both sleep diary and actigraph measurements [17] and have shown acceptable relative validity for use in large studies of adolescents. All questionnaires used in this study were pilot tested in the sample population before use in this study to ensure they were suitable for use in OSSLS2.

If the errors in self-reported sleep data are non-differential then this would lead to attenuation of results, rather than overestimation of relationships [34]. Similar relationships between adolescent sleep duration and body composition measures have been seen in studies using self or parental reported sleep duration compared to those using objective measures based on actigraphy [6,35,36]. Reported sleep times and the shorter sleep times for boys found in this cohort are similar to those found in a recent meta-analyses of adolescents from 23 countries [12], where adolescents aged fifteen to eighteen years slept for around 9 to 10 hours each day. The amount of catch-up sleep reported at weekends is very similar to the 40 minutes reported in the only other large-scale study of sleep in New Zealand adolescents [37]. We also had no measure of participants’ pubertal status, as it was not feasible to collect accurate information on this within the current study design, but as the age of participants ranged from fifteen to eighteen years of age, it is likely that the majority of participants were pubertal or post-pubertal. We did not collect information on depression, which has been shown to play a role in the relationship between sleep and body composition [5].

The use of BIA measurements is not without limitations. Estimates of fat and fat-free mass are calculated using sex and aged based equations [38]. Because of the nature of carrying out school-based research, particularly the logistical demands, in samples of this size, BIA measurements were carried out throughout the school day. Therefore BIA estimates may be affected by recent exercise, food consumption or hydration status.

However, it would be impractical to use other measures such as DXA in large population studies and fat mass and fat-free mass estimates obtained from BIA measurements in New Zealand adolescents have been found to be highly correlated with DXA measurements [22]. The use of standardised operating procedures for body composition measuring, intensive training in measurement, and quality control procedures in place during the study would ensure that this error is minimised.

One strength of this study is the use of an FFQ that has been developed and validated for use specifically in this study. This means that the food data collected is robust [17]. However, as the FFQ used is non-quantitative, we can only be sure that frequency of consumption of foods does not play a part in these relationships and the role of particular foods cannot be assessed independently of energy intake. While using more intensive methods such as food diaries would provide data on nutrients and energy intake, compliance with such methods in this age group is low, and results of our pretesting showed that almost 50% of adolescents failed to complete a four day food record [17]. Another limitation of the current study is that we were unable to adjust for physical activity in these analyses. Therefore we cannot rule out the possibility that energy imbalance, rather than food choice itself, is an important moderator in these relationships, as has been found in previous studies [29,36], or that energy balance may be a mediator of the sleep-obesity pathway. The 78% participation rate in OSSLS2 shows that the current sample is representative of the Otago region but it is not nationally representative due to the low prevalence of those of Māori or Pacific origin, who are also more likely to be of lower socio-economic status and to have higher BMIs [2].

In conclusion, the relationship between shorter sleep duration in boys and a more detrimental body composition, both in terms of lean and fat mass, indicates that sex specific factors may play a role in this relationship in older adolescents. Despite the fact that the study population may not be nationally representative and that the majority of the data was collected by questionnaire, our results reflect international results, including results from studies using more intensive data collection methods. Further research is needed to identify what sex specific factors are responsible for these findings so that targeted approaches to help reduce rates of overweight and obesity could be identified.

## Abbreviations

OSSLS2: Otago School Students Lifestyle Survey Two; NZEO: New Zealand European or other; SES: Socio-economic status; NZDep06: New Zealand deprivation index score; WC: Waist circumference; BIA: Bio-electrical impedance analysis; FMI: Fat mass index; FFMI: Fat-free mass index; WHtR: Waist-to-height ratio; DXA: Duel-energy X-ray absorbance.

## Competing interests

The authors declared that they have no competing interests.

## Authors’ contributions

ASH and PMLS were responsible for conception of this particular study and performing data analysis. PMLS drafted the initial manuscript. PMLS and KEB are the principal investigators for the overall project and were responsible for conception and design of the project and oversaw questionnaire design, data collection and processing. ASH, JEW and AL contributed to the design of the project, including questionnaire design, and data collection and processing. MAP contributed to data collection and processing. SMW contributed to the design of the project and oversaw statistical analyses. All authors were involved in writing the paper and have read and approved the final version of the manuscript.
